# Effectiveness of Group vs. Individual Therapy to Decrease Peer Problems and Increase Prosociality in Children

**DOI:** 10.3390/ijerph18083950

**Published:** 2021-04-09

**Authors:** Silvia Melero, Alexandra Morales, José P. Espada, Xavier Méndez, Mireia Orgilés

**Affiliations:** 1 Department of Health Psychology, Miguel Hernández University, 03202 Elche, Spain; alexandra.moraless@umh.es (A.M.); jpespada@umh.es (J.P.E.); morgiles@umh.es (M.O.); 2 Department of Personality, Assessment and Psychological Treatment, University of Murcia, 30100 Murcia, Spain; xmendezc@um.es

**Keywords:** transdiagnostic, Super Skills for Life, peer problems, prosocial behavior, children

## Abstract

Emotional difficulties in children are often shown to be associated with peer problems and low prosocial behaviors. Super Skills for Life (SSL) is a transdiagnostic protocol for the prevention of emotional problems in children and has also obtained improvements of other comorbid symptoms. This study aimed at comparing the effects of SSL in reducing peer problems and increasing prosocial behaviors in children aged 8 to 12 years between the group and the individual modalities. For this purpose, 140 children (35% girls) received the program, 70 in group format and 70 in individual format, and were evaluated at the baseline, posttest, and after one year. Both modalities were effective in enhancing social relationships in children, although the individual modality showed more promising results. Children belonging to the individual modality group presented fewer peer problems (less social isolation and rejection, greater social acceptance, more friends) and greater prosocial behaviors (helping, empathy, kindness, and sharing) compared to children receiving the therapy in group modality, both in the short and in the long term. In conclusion, this study provides evidence of SSL protocol efficacy for improving children’s peer relationships and prosocial behaviors and encourages the implementation of transdiagnostic interventions in both clinical and educational settings.

## 1. Introduction

Prosocial behavior is evident in humans from a young age; thus, infants between 12 and 24 months of age begin to show positive other-oriented behaviors such as helping, sharing, or comforting [1]. During early childhood, sociomoral, cognitive, and emotional development allows children to act prosocially based on the needs of their peers, with whom they maintain a reciprocal relationship [2,3]. However, at these age, children tend to favor themselves when sharing, helping, or cooperating with others, up to around 7 years of age, when sharing becomes more equal, and they develop greater empathy for others [3,4,5]. These prosocial behaviors not only contribute to better social relationships but also are affectively rewarding for the benefactors [6,7,8]. Thus, prosocial behavior would be associated with positive mood and, in turn, with the maintenance of this behavioral pattern in the future [4].

During middle and late childhood, the feeling of belonging within a peer group also becomes particularly important [9]. Young people are therefore motivated to act prosocially, in order to find and maintain their place among their peers [7]. Prosociality increases peer acceptance, which is related to better academic and social outcomes, and reduces the likelihood of being rejected or bullied [10,11]. Furthermore, studies evidence that a high pattern of prosocial behavior is predictive of future positive social adjustment and a protective factor against internalized problems [7,10]. Likewise, this relationship has been found inversely, since the internalized problems in children were predictors of greater peer problems (e.g., victimization, poor social skills, and withdrawal) and lower prosocial behaviors [12,13,14]. Therefore, the need to promote these positive behaviors during childhood is evident, especially in the school setting [11].

Children with anxiety and depressive symptoms are often inhibited, sensitive, have few friends, and show poor confidence and social skills to cope with peer bullying. For this reason, prevention programs are recommended to reduce their vulnerability to be victimized and developing more severe emotional disorders [15]. Existing interventions are consistent in incorporating social skills training, improvement of self-esteem and social performance, exposure, and reduction of emotional symptoms [15,16]. Transdiagnostic interventions are useful to address these problems, as they include different effective techniques targeting the underlying mechanisms common to various difficulties in a single protocol [17,18]. Currently existing transdiagnostic prevention programs for schoolchildren do not include social skills training or are specifically targeted at victims of bullying [15,19]. In addition, these protocols were designed to be applied in a group modality, which is not always feasible. Although group-based programs are cost- and time-effective and allow for interaction with and feedback from others, they may inadvertently promote avoidance behaviors and negative peer modeling [20,21]. Therefore, implementing individual interventions may be more effective in training skills and behaviors, adapting them to the characteristics of each child [21,22,23].

Super Skills for Life (SSL) is the first transdiagnostic program for the indicated prevention of children’s emotional problems that can be applied in both group and one-to-one formats [24]. This protocol based on Cognitive Behavioral Therapy (CBT) is comprised of components such as emotional regulation, relaxation, cognitive reappraisal, problem-solving and includes behavioral activation, social skills training, and self-monitoring [17,24]. The SSL transdiagnostic program addresses core risk factors for different disorders (such as low self-esteem, poor social skills, cognitive dysfunction) and provides strategies for coping with stress-provoking situations and social conflicts. Moreover, it is worth mentioning the enhancement of social competence in children through the learning of social skills—both verbal and non-verbal—and the improvement of their self-perception through video-feedback with cognitive preparation. Several studies have reported the value of these techniques to increase children’s social competence and prosocial behaviors and reduce anxiety behaviors in social interactions [12,17,25,26]. Social competence allows children to better manage interpersonal conflicts and therefore be less rejected and more well-liked within their social group, which is a protective factor against other psychological difficulties [27,28].

Both the original English and the Spanish version of SSL have shown efficacy in reducing emotional symptoms (i.e., anxiety and depression) in children aged 8–12 years and have also promoted improvements of other externalizing difficulties [17,29,30]. Specifically, the group version obtained significant improvements in children with hyperactivity/inattention and behavioral and peer problems in the long term [17,30], while the individual version showed these benefits in the short term [29]. In addition, both versions have offered social benefits to the participating children, improving their social performance and interaction skills [25,26]. SSL in group format allowed the children to perform in front of their peers and practice skills together, while the one-to-one format provided more emphasis on each child’s needs. Thus, both versions have shown to be effective in reducing psychological difficulties and increasing children’s strengths [17,29,30]. However, no studies have been conducted comparing the results obtained with the two formats.

In light of this, the present study specifically aimed at comparing the effects of SSL to reduce peer problems and increase prosocial behaviors in children aged 8 to 12 years between the group therapy and the individual therapy modalities of the program. To this end, these variables were assessed in children before and after receiving the intervention, as well as one year later. Based on previous studies and considering the evidence obtained regarding the SSL program, it was hypothesized that: (1) the individual modality will obtain significantly greater improvements than the group modality, due to greater intervention customization, practice opportunities, and therapist feedback [21,23,29]; (2) regardless of the application format, children will reduce peer problems and increase their prosocial behaviors after receiving the program and one year later [17,29,30].

## 2. Materials and Methods

### 2.1. Participants

The sample of this study consisted of 140 children aged 8 to 12 years (*M* = 9.48; *SD* = 1.26); 35% of these children were girls. The participants were mostly of Spanish origin (95.7%), residing in central and southeastern Spain. The rest were born in other countries such as the United Kingdom, Brazil, and Poland but were fluent in Spanish. The children belonged to 37 public, private, and charter schools. In general, the children had one sibling on average. Table 1 provides details of the socio-demographic characteristics of the children.

The study was introduced to families of children in the 2nd–6th grade of primary school. To participate, parents had to complete a screening assessment through an online form. Those families who reported emotional symptoms in their children were invited to participate in the study. To control for possible extraneous variables that could affect the conclusions of the study, the following exclusion criteria were established: (1) presenting a severe developmental problem and (2) be receiving psychological/pharmacological treatment for emotional and/or behavioral problems at that time.

### 2.2. Measures

#### 2.2.1. Sociodemographic Variables

Data were collected on age, gender, nationality, and number of siblings of the participant children.

#### 2.2.2. Self-Report Assessment

The Strengths and Difficulties Questionnaire–Child version (SDQ-S) [31] is a brief self-report questionnaire for assessing children and adolescents’ psychological adjustment. This screening tool is composed of 25 items and comprises 5 scales of 5 items each: Emotional Symptoms, Conduct Problems, Hyperactivity/Inattention, Peer Problems, and Prosocial Behavior. Items are rated on a 3-point scale ranging from 0 (*Not true*) to 2 (*Certainly true*). The Total difficulties score is obtained by summing all subscales, except for the Prosocial behavior subscale (scores range from 0 to 40). In the current study, only the Peer problems and Prosocial Behavior subscales were used for the analyses. Both have a range of scores from 0 to 10, but higher scores indicate more severity in the subscale Peer problems and more positive behaviors in Prosocial behavior. The SDQ Spanish version has demonstrated satisfactory psychometric properties, with adequate levels of reliability for the total score (0.84) and its subscales (0.71–0.75) [32]. In this study, the ordinal alphas were 0.70 for the Peer problems subscale and 0.69 for the Prosocial behavior subscale.

#### 2.2.3. Parental Assessment

The Strengths and Difficulties Questionnaire–Parent version (SDQ-P) [31] has the same scales and number of items as the self-reported version. In this study, only the Emotional Symptoms score was analyzed for selection purposes. Families who reached or exceeded the cut-off point of 4 on this subscale were invited to participate, as it indicates the presence of anxious and depressive symptoms in children. The Emotional Symptoms subscale showed a Cronbach alpha coefficient of 0.71 and of 0.76 for the Total score in the Spanish SDQ-P [33]. In the current study, ordinal alpha of the Emotional Symptoms subscale was moderate (α = 0.58) [34].

### 2.3. Procedure

The Ethics Committee of Miguel Hernández University (Spain) approved the study (DPS.MO.01.17). In order to adequately represent the socioeconomic structure of the Spanish population, the study was disseminated in two ways. The collaboration of 14 schools was requested to send a letter to the families of schoolchildren aged 8–12 years informing them about the study. Moreover, the research was promoted through social media. The families voluntarily completed an online form about their children’s emotional well-being. After analyzing the results, the intervention was offered to children who presented emotional problems as reported by their parents. Meetings were held to provide information to parents about the characteristics of the intervention and the confidentiality of their data and to request informed written consent.

**Figure 1:**
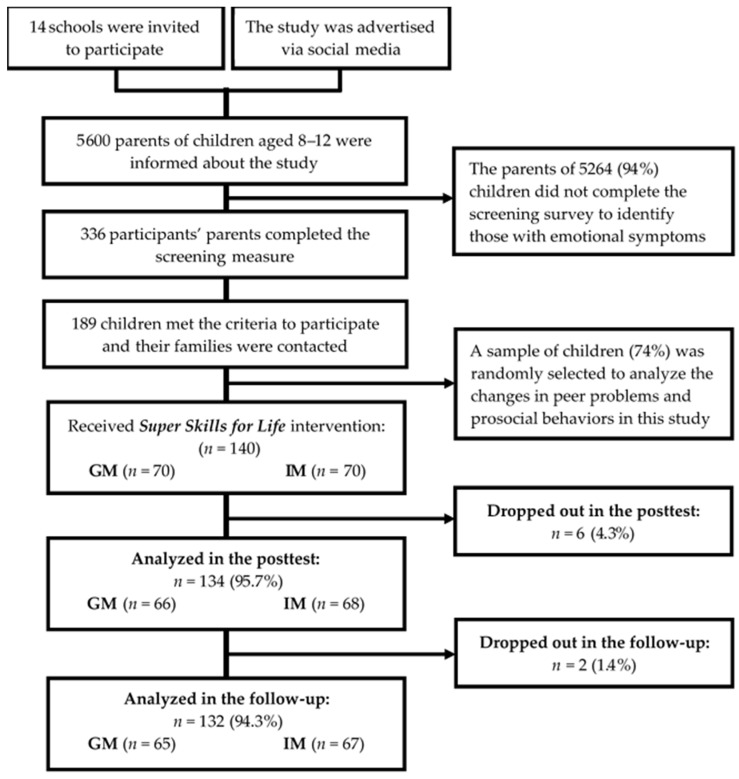
Progress of children participating in the trial. GM = Group Modality; IM = Intervention Modality.

The study was conducted following a quasi-experimental research model with three repeated measures (pretest, posttest, and follow-up) for the variables evaluated. The research procedure and flow of children’s participation in the trial is shown in the Figure 1. The 140 participating children were assessed at baseline (pretest), 134 children after completion of the intervention (posttest), and 132 one year later (follow-up). The children’s data were collected online with the assistance of the facilitators. The sample was divided into two experimental conditions: 70 children received the intervention in group modality and the other 70 in individual modality. All children attended the program sessions after school hours. The program was implemented by 18 psychologists (both genders) with a master’s degree in child and adolescent psychology and at least 2 years of clinical practice. Facilitators were instructed on the implementation of the program in a one-day workshop. The researchers responsible for the study held weekly meetings with the facilitators to provide them with all the necessary materials and supervise the correct application of the protocol.

### 2.4. Intervention Modalities

The Super Skills for Life program was designed by the researchers Essau and Ollendik [24] for the indicated prevention of emotional problems in children. The intervention is composed of 8 sessions in which children learn to manage their emotions, detect and modify negative thoughts, relax, expose themselves to anxiety-provoking situations, interact with others, and solve problems. This transdiagnostic protocol was subsequently translated and adapted for the Spanish population, both for the group and the individual application [29,30]. Both versions maintain the same contents, but the implementation methodology is different. In the group version, the program was delivered to small groups of 6–8 children, who received feedback from the therapist as well as from their peers and interacted with other children. On the other hand, in the individual format, the exposure and role-playing activities involved only the therapist and the child, allowing greater customization of the intervention. Table 2 shows the main differences between the two versions.

The SSL program was delivered over eight 45-minute sessions, once a week, in both modalities. The children received a notebook at the beginning of the program to complete exercises, readings, games, and role-playing to learn the skills. In addition, they were required to complete a homework assignment to practice and reinforce what they had learned. Families were kept informed of their children’s progress and the contents covered during the program via email. However, the individual version also allowed for more direct feedback to parents. At the end of the program, all families received a report on the changes produced in their children after participating in the intervention.

### 2.5. Data Analysis

Analyses were performed using SPSS v25. Baseline equivalence between the therapy conditions in sociodemographic variables and main outcomes (peer problems and prosocial behavior) was analyzed using Student’s t-test and cross-tabulation for quantitative and qualitative variables, respectively. Cohen’s *d* was calculated as a measure of effect size [35]. Attrition analyses were conducted to identify the profile of participants lost in every assessment (posttest and 1-year follow-up) from the group therapy modality (*n* = 4 in the posttest and *n* = 5 in the follow-up) and the individual therapy modality (*n* = 2 in the posttest and *n* = 3 in the follow-up). The effects of SSL to reduce peer problems and increase prosocial behavior in both therapy modalities were explored using generalized estimating equations (GEE), adjusting for the baseline assessment, children’s age and sex, and clustering within schools. GEE is a widely used statistical procedure for evaluating the efficacy of interventions, both in randomized control trials and in cluster-randomized control trials. GEE increases the statistical power when the study sample is small and allows estimating changes in main outcomes over time and the use of incomplete data, without the need to eliminate data from the analyses [36]. Effects of the intervention were tested by comparing both therapy conditions at posttest and after 1 year of follow-up (intergroup evaluation) and by comparing the three measures for each therapy condition: pretest, posttest, and 1-year follow-up (intragroup evaluation). Because of the ordinal nature of the scales, the ordinal alphas of the subscales with the current sample were calculated using *R* Studio v1.3.1093.

## 3. Results

### 3.1. Attrition Analysis

Regarding external validity, no differences were found in children’s age (*p* = 0.67) or sex (*p* = 0.49), peer problems (*p* = 0.62), and prosocial behavior (*p* = 0.65) baseline scores between children who were lost to follow-up at posttest and those who provided their evaluation at posttest and 1 year later. At the 1-year follow-up, no differences were found in children’s age (*p* = 0.31) or sex (*p* = 0.36), peer problems (*p* = 0.44), and prosocial behavior baseline scores (*p* = 0.23) between children who were lost to 1-year follow-up and those who provided their evaluation. Regarding internal validity, no differences were found in the retention rate of children at posttest (*p* = 0.41) and at 1-year follow-up between the two therapy conditions (*p* = 0.47). 

On average, children’s attendance to program sessions was high (*M*_sessions attended_ = 7.57; *SD* = 1.21), and differences were found between the two conditions, *t* = −4.35, *p* ≤ 0.001; *d* = 0.74). Children receiving the individual therapy modality attended all sessions, meanwhile those receiving the group therapy modality attended a mean of 7.15 sessions (*SD* = 0.19).

At baseline, both conditions were equivalent in terms of sociodemographic variables—including children’s sex (*p* = 0.11) and age (*p* = 0.31), nationality (*p* = 0.40), and number of siblings (*p* = 0.18)—and the main outcomes, i.e., peer problems (*p* = 0.78) and prosocial behavior (*p* = 0.41) (Table 1).

### 3.2. Intergroup Assessment

At posttest, children receiving the individual therapy presented lower score in peer problems and higher scores in prosocial behavior, compared to children receiving the group therapy. At 1-year follow-up, children belonging to the individual therapy group presented a lower score in peer problems and a higher score in prosocial behavior, compared to children receiving the group therapy (Table 3). 

### 3.3. Group Therapy Modality

**Figure 2:**
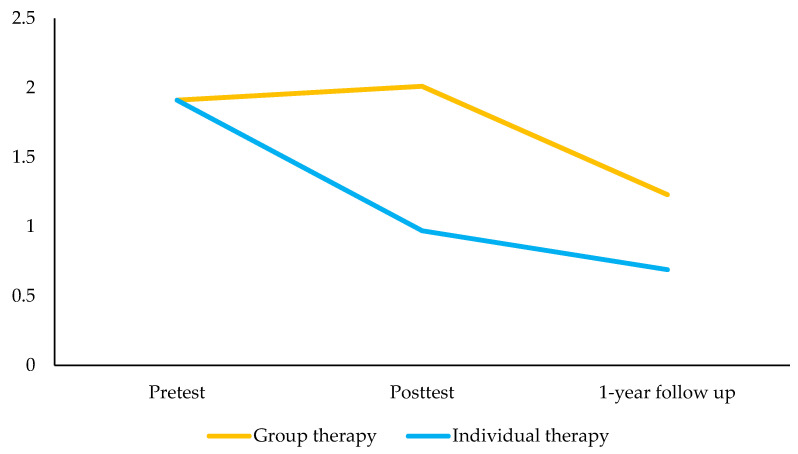
Estimated marginal means of the peer problems score between pretest, posttest, and 12-months follow-up by therapy condition.

**Figure 3:**
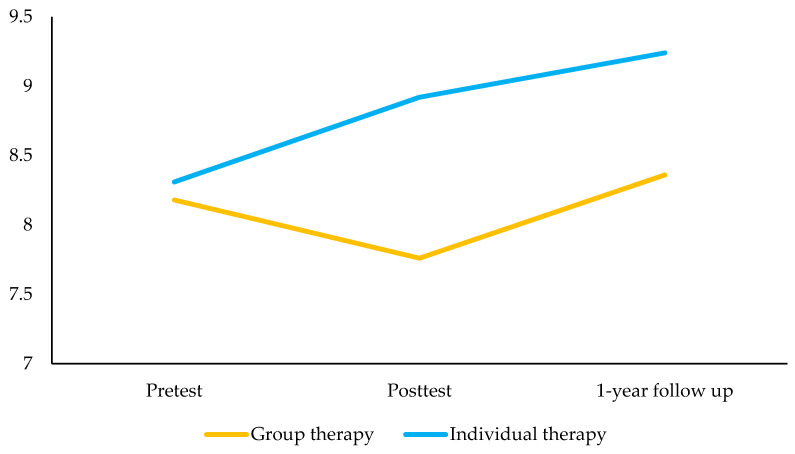
Estimated marginal means of the prosocial behavior score between pretest, posttest, and 12-months follow-up by therapy condition.

No statistically significant changes were observed in peer problems and prosocial behavior between pretest and posttest in children receiving the group therapy modality. Compared to the pretest, children receiving the group therapy modality presented lower peer problems 1-year post-intervention (Table 3; Figure 2). The intervention did not have a statistically significant impact on prosocial behavior 1-year post-intervention, although the trend was to increase prosocial behaviors (Figure 3).

### 3.4. Individual Therapy Modality

Compared to the pretest, children belonging to the individual therapy modality group presented lower peer problems and higher prosocial behavior at posttest (Table 3). Compared to the pretest, children receiving the individual therapy modality presented lower peer problems and higher prosocial behavior 1-year post-intervention (Figure 2; Figure 3).

### 3.5. Differences between Modalities

Table 4 shows the differences between the two modalities in each of the items analyzed. Regarding peer problems, significant differences were obtained in the posttest for items 6, 11, and 14. This indicates that the children who participated in the individual version of the program played alone to a lesser extent, had at least one friend, and tended to be liked better by their peers, compared to the children who received the program in group format. At follow-up, a significant difference was also found for item 14, whereby children who participated in the one-to-one version of the program were more accepted by their peers. 

The one-to-one version also promoted more significant improvements in prosocial behaviors than the group version. Specifically, the children showed greater empathy for others’ feelings (item 1), shared (item 4), and helped (item 9) to a greater extent, both at posttest and at one-year follow-up. Children in the individual format group also showed greater kindness to other young children after completing the program (item 17) and were more volunteering to help others one year later (item 20).

## 4. Discussion

Emotional difficulties in children are often shown to be associated with peer problems, and both problems may have parallel trajectories from childhood to adolescence [12,13,14,37,38]. In addition, this pattern of symptoms is accompanied by a lower prosocial behavioral repertoire [13,27,37]. Since the Super Skills for Life program is targeted primarily at emotional problems, it was expected to have a positive impact on the improvement of peer problems and prosocial behaviors [17,29,30]. Furthermore, the social skills training and video-feedback with cognitive preparation components enabled children to learn more prosocial manners of relating to others [17,25,26]. Hence, the present study was aimed at evaluating the efficacy of the transdiagnostic SSL program in reducing peer problems and increasing prosocial behaviors, comparing the group and individual modalities. For this purpose, a sample of schoolchildren was assigned to the group modality, and another sample to the individual modality, and the outcomes of both formats were compared. Consistent with our initial hypothesis, children who received SSL in a one-to-one version showed fewer peer problems and greater prosocial behaviors compared to the group modality participants, both in the short and in the long term. 

As expected, the individualization of the intervention and the tailoring to each child’s needs led to more significant achievements in the social interaction variables analyzed, compared to the group application [21,22,23]. During the program sessions, every child was able to practice to a greater extent those behaviors in which he/she presented difficulties, obtaining direct modeling and feedback from his/her therapist [29]. Using their own examples, they were provided with strategies for coping with social conflicts, resulting in less social isolation and rejection, greater social acceptance, and more friends upon completion of the program. They also reported behaving more prosocially, caring about others’ feelings, being kind, sharing, and helping to a greater extent. These changes were maintained at follow-up, with greater social acceptance and willingness to help voluntarily being observed in the children receiving the individual modality therapy. These results provide evidence of the efficacy of individual versus group interventions in the acquisition of protective skills and behaviors against the risk of experiencing social problems in emotionally vulnerable children [22,23,25].

Our second hypothesis suggested that children who received the SSL intervention would reduce their peer problems and enhance prosocial behaviors at posttest and follow-up, regardless of the application format. Altogether, the participating children reported positive outcomes after receiving the SSL program, but in contrast to previous studies, the program implementation modality influenced the outcomes obtained [20,21]. The group format did not achieve significant improvements at posttest on the evaluated variables. However, significantly lower scores on peer problems and a tendency to increase prosocial behaviors were found at one-year follow-up. Previous studies of the program also found that improvements in secondary outcomes were obtained in the long term [17,30]. These results could be explained because one year later the children had more opportunities to show prosocial behaviors and generalize them in their different contexts [17,30]. Although group interventions have advantages (e.g., time saving, peer interaction, multiple feedback), their implementation poses challenges for facilitators in managing potential conflicts between children, limit setting, and adjusting the application pace [39]. Furthermore, children may have more opportunities for avoidance in a group and for missing content if they do not attend some sessions [20,39]. In our case, the children in the group format attended an average of 7.15 sessions, which may have influenced the difference in results with respect to the individual version (full attendance).

As discussed above, the display of prosocial behaviors in childhood promotes greater reciprocity and peer acceptance and better future psychological adjustment [7,10,11]. Consequently, these types of transdiagnostic interventions targeting shared risk factors among different problem areas, such as social relationships, are quite beneficial for a heterogeneous group of children [15,17,18,19]. Therefore, the implementation of transdiagnostic programs such as the SSL is an effective strategy both to avoid social difficulties in children with emotional symptoms and to prevent the emergence of more severe internalizing problems in children with social deficits [15,17,30]. Our results revealed that both the group and the individual modalities were effective in enhancing social relationships in children, although the intervention had a higher impact when working with children one to one. Thus, this study has important research and clinical implications because, in addition to providing evidence of SSL protocol efficacy for improving children’s peer relationships and prosocial behaviors, it guides professionals for targeting the design and application to schoolchildren with these difficulties.

Despite the positive findings of the study, the conclusions should be interpreted with caution due to certain limitations. First, only self-reports were administered to measure the changes produced at the three time points. Future research should examine whether these results are replicated from the perspective of other adults close to the children, such as parents or teachers. Second, this is preliminary research with a small sample of children, hence it would be interesting to carry out the study with larger samples for a better generalization of the results. Finally, future studies should aim to explore the mediating or moderating factors of the program’s efficacy influencing the social variables evaluated. Furthermore, since the implementation of individualized protocols may be more challenging, especially in some contexts such as schools, future research could aim at adapting the program using a blended approach. That is, the SSL program could be applied in a group format but include some individual sessions to ensure that each of the children has acquired the appropriate skills. For example, social skills could be trained one to one, and once acquired, the children would practice them with the rest of the group [17,25,26].

## 5. Conclusions

Considering these aspects, the strength of this study is that it is the first to compare the effectiveness of the two modalities of the SSL program in the short and long term regarding children’s social relationships and behaviors. According to our results and until further research on the use of both modalities is completed, we recommend using the one-to-one version of SSL for enhancing prosocial behaviors and peer problems in schoolchildren. In conclusion, this study contributes to the promotion of psychosocial well-being in childhood through a transdiagnostic approach and encourages the implementation of effective interventions in both clinical and educational settings [15,17,18,19].

## Figures and Tables

**Table 1:** Sociodemographic characteristics at baseline of participating children by intervention modality.

Characteristics	Group Therapy (*n* = 70)	Individual Therapy (*n* = 70)	Total (*n* = 140)	*p*-Value
Female, *N* (%)	20 (28.6)	29 (41.4)	49 (35)	0.11
Mean age (*SD*), years	9.59 (1.32)	9.37 (1.20)	9.48 (1.26)	0.24
Nationality, *N* (%)				
Spanish	68 (97.1)	66 (94.3)	134 (95.7)	0.40
Other	2 (2.9)	4 (5.7)	6 (4.3)	
Mean number (*SD*) of siblings	1.06 (0.50)	0.93 (0.62)	0.99 (0.56)	0.18

**Table 2:** Differences between the Super Skills for Life (SSL) program modalities.

Session	Group	Individual
1	Children must introduce themselves in the group and interact with the other children.	Child and therapist introduce themselves by simulating a television interview.
2	Children take turns playing at recognizing emotions in their peers. Recordings of children’s performance are watched by the children and their peers.	Emotion recognition games are played only by therapist and child. Recordings of children’s performance are only watched by the child. The child assesses his/her own social performance.
3	Examples of the most common fears and concerns in children are discussed.	Examples are adapted to the child’s fears and concerns.
4	The relationship between thoughts, emotions, and behavior is explained through examples of usual situations.	Situations experienced by the child are discussed for greater transference into the natural setting.
5	Relaxation strategies are taught in a group, with everyone participating at the same time.	Relaxation strategies are taught to the child alone, allowing for better concentration.
6	Role-playing activities are performed together by the children. The videotaped activity consists of joining a group.	Role-playing activities are practiced by child and therapist. The videotaped activity consists of meeting someone new (the child should approach and talk to an unknown person).
7	Recordings are watched by the children and their peers.	Recordings are only watched by the child. The child assesses his/her own social performance.
8	Children review the skills learned during the program in front of their peers.	The child reviews the skills learned and checks his/her progress during the program through the videos.

**Table 3:** Generalized estimating equations (GEE) for repeated measures and effect size estimates for the intervention effect on outcomes in the posttest and 12-month follow-up (compared to the baseline) and by therapy modality.

Outcomes	Sample	Post-Treatment		12-Month Follow-Up	
		AOR (95% CI)	*p* Value	AOR (95% CI)	*p* Value
Peer problems	Group therapy (intragroup)	1.10 (0.69, 1.74)	0.67	0.50 (0.33, 0.75)	0.001
	Individual therapy (intragroup)	0.39 (0.24, 0.63)	≤0.001	0.29 (0.19, 0.45)	≤0.001
	Total (intergroup)	2.76 (1.64, 4.67)	≤0.001	1.70 (1.10, 2.63)	0.01
Prosocial behavior	Group therapy (intragroup)	0.65 (0.42, 1)	0.06	1.19 (0.72, 1.94)	0.48
	Individual therapy (intragroup)	1.84 (1.24, 2.73)	0.002	2.51 (1,67, 3.79)	≤0.001
	Total (intergroup)	0.27 (0.15, 0.48)	≤0.001	0.42 (0.24, 0.72)	0.002

AOR = Adjusted Odds Ratio. CI = Confidence Interval. Higher scores denote greater symptomatology, except for Prosocial behavior (higher scores indicate more prosocial behaviors). Each analysis was adjusted for baseline assessment, children’s age and sex, and clustering within schools.

**Table 4:** Comparison of items from peer problems and prosocial behavior subscales by time and by intervention modality.

	Time	Group Therapy (*n* = 70)	Individual Therapy (*n* = 70)	Total (*n* = 140)	*p*-Value	*d*
**Peer problems**						
ITEM 6: Rather solitary, tends to play alone *(I am usually on my own)*	Pretest	0.38 (0.62)	0.43 (0.73)	0.46 (0.68)	0.65	-
	Posttest	0.56 (0.75)	0.10 (0.42)	0.33 (0.64)	≤0.001	0.75
	1 year	0.24 (0.49)	0.09 (0.37)	0.16 (0.44)	0.057	-
ITEM 11: Has at least one good friend *(I have one good friend or more)*	Pretest	0.18 (0.54)	0.14 (0.46)	0.16 (0.50)	0.71	-
	Posttest	0.22 (0.54)	0.06 (0.23)	0.14 (0.42)	0.03	0.38
	1 year	0.10 (0.39)	0.03 (0.24)	0.06 (0.32)	0.25	-
ITEM 14: Generally liked by other children *(Other people my age generally like me)*	Pretest	0.35 (0.51)	0.33 (0.56)	0.34 (0.53)	0.83	-
	Posttest	0.39 (0.58)	0.15 (0.39)	0.27 (0.50)	0.005	0.48
	1 year	0.25 (0.53)	0.07 (0.26)	0.16 (0.42)	0.01	0.43
ITEM 19: Picked on or bullied by other children *(Other children or young people pick on me)*	Pretest	0.49 (0.65)	0.46 (0.69)	0.47 (0.67)	0.85	-
	Posttest	0.42 (0.63)	0.29 (0.54)	0.36 (0.59)	0.21	-
	1 year	0.27 (0.60)	0.24 (0.58)	0.25 (0.58)	0.76	-
ITEM 23: Gets on better with adults than with other children *(I get on better with adults than with people my age)*	Pretest	0.54 (0.67)	0.52 (0.65)	0.53 (0.66)	0.84	-
	Posttest	0.52 (0.71)	0.37 (0.66)	0.44 (0.69)	0.22	-
	1 year	0.37 (0.63)	0.22 (0.54)	0.29 (0.59)	0.17	-
**Prosocial behavior**						
ITEM 1: Considerate of other people’s feelings *(I try to be nice to other people)*	Pretest	1.59 (0.62)	1.62 (0.57)	1.61 (0.59)	0.73	-
	Posttest	1.58 (0.58)	1.81 (0.46)	1.70 (0.53)	0.01	0.43
	1 year	1.67 (0.50)	1.88 (0.37)	1.78 (0.45)	0.007	0.47
ITEM 4: Shares readily with other children *(I usually share with others)*	Pretest	1.62 (0.57)	1.65 (0.53)	1.64 (0.55)	0.71	-
	Posttest	1.42 (0.70)	1.68 (0.53)	1.55 (0.63)	0.02	0.41
	1 year	1.57 (0.68)	1.75 (0.47)	1.66 (0.59)	0.09	0.30
ITEM 9: Helpful if someone is hurt *(I am helpful is someone is hurt)*	Pretest	1.71 (0.45)	1.61 (0.57)	1.66 (0.52)	0.27	-
	Posttest	1.55 (0.64)	1.84 (0.44)	1.70 (0.56)	0.003	0.52
	1 year	1.65 (0.57)	1.90 (0.30)	1.78 (0.47)	0.003	0.54
ITEM 17: Kind to younger children *(I am kind to younger children)*	Pretest	1.74 (0.53)	1.86 (0.46)	1.80 (0.50)	0.16	-
	Posttest	1.64 (0.62)	1.91 (0.33)	1.78 (0.51)	0.002	0.54
	1 year	1.90 (0.39)	1.94 (0.34)	1.92 (0.36)	0.58	-
ITEM 20: Often volunteers to help others *(I often volunteer to help others)*	Pretest	1.50 (0.63)	1.62 (0.54)	1.56 (0.59)	0.22	-
	Posttest	1.55 (0.56)	1.72 (0.48)	1.64 (0.52)	0.059	-
	1 year	1.52 (0.66)	1.79 (0.41)	1.66 (0.56)	0.007	0.49

*d* = Cohen’s *d* effect size. *Note*: Items 11 and 14 are inverse, thus the higher the score, the greater the peer problems.

## Data Availability

The data that support the findings of this study are available on request from the corresponding author. The data are not publicly available due to privacy or ethical restrictions.
